# PastoCovac and PastoCovac Plus as protein subunit COVID-19 vaccines led to great humoral immune responses in BBIP-CorV immunized individuals

**DOI:** 10.1038/s41598-023-35147-y

**Published:** 2023-05-18

**Authors:** Amitis Ramezani, Rahim Sorouri, Saiedeh Haji Maghsoudi, Sarah Dahmardeh, Delaram Doroud, Mona Sadat Larijani, Sana Eybpoosh, Ehsan Mostafavi, Alireza Olyaeemanesh, Mostafa Salehi-Vaziri, Anahita Bavand, Ghazaleh Zarghani, Ladan Moradi, Fatemeh Ashrafian, Fahimeh Bagheri Amiri, Parisa Mashayekhi, Zahra Tahmasebi, Alireza Biglari

**Affiliations:** 1 Clinical Research Department, Pasteur Institute of Iran, No 69, Pasteur Ave., Tehran, Iran; 2 IPI Directorate, Pasteur Institute of Iran, Tehran, Iran; 3 Modeling in Health Research Center, Institute for Futures Studies in Health, Kerman University of Medical Sciences, Kerman, Iran; 4 Department of Biostatistics and Epidemiology, School of Public Health, Kerman University of Medical Sciences, Kerman, Iran; 5 Department of Vaccination, Pasteur Institute of Iran, Tehran, Iran; 6 Quality Control Department, Production and Research Complex, Pasteur Institute of Iran, Tehran, Iran; 7 Department of Epidemiology and Biostatistics, Research Centre for Emerging and Reemerging Infectious Diseases, Pasteur Institute of Iran, Tehran, Iran; 8 Health Equity Research Centre and National Institute of Health Research, Tehran University of Medical Science, Tehran, Iran; 9 COVID-19 National Reference Laboratory, Pasteur Institute of Iran, Tehran, Iran; 10 Department of Molecular Medicine, Biotechnology Research Center, Pasteur Institute of Iran, Tehran, Iran; 11 School of Medicine, Tehran University of Medical Sciences, Enghelab Street, P.O. BOX 14155-6559, Tehran, Iran

**Keywords:** Health care, Diseases, Infectious diseases, Drug delivery

## Abstract

The optimal booster vaccine schedule against COVID-19 is still being explored. The present study aimed at assessment of the immunogenicity and antibody persistency of inactivated-virus based vaccine, BBIP-CorV and protein-subunit based vaccines, PastoCovac/Plus through heterologous and homologous prime-boost vaccination. Totally, 214 individuals who were previously primed with BBIBP-CorV vaccines were divided into three arms on their choice as heterologous regimens BBIBP-CorV/PastoCovac (n = 68), BBIBP-CorV/PastoCovac Plus (n = 72) and homologous BBIBP-CorV (n = 74). PastoCovac booster recipients achieved the highest rate of anti-Spike IgG titer rise with a fourfold rise in 50% of the group. Anti-RBD IgG and neutralizing antibody mean rise and fold rise were almost similar between the PastoCovac and PastoCovac Plus booster receivers. The antibody durability results indicated that the generated antibodies were persistent until day 180 in all three groups. Nevertheless, a higher rate of antibody titer was seen in the heterologous regimen compared to BBIP-CorV group. Furthermore, no serious adverse event was recorded. The protein subunit-based booster led to a stronger humoral immune response in comparison with the BBIP-CorV booster receivers. Both the protein subunit boosters neutralized SARS-CoV-2 significantly more than BBIP-CorV. Notably, PastoCovac protein subunit-based vaccine could be successfully applied as a booster with convenient immunogenicity and safety profile.

## Introduction

SARS-CoV-2 has still remained an unsolved medical matter due to the variants with the potency of immune escape and waning of elicited immunity either natural or vaccine-induced, leading to a growing number of vaccine-breakthrough incidents and re-infections globally^1–4^. Mass vaccination programs through different platforms are held with the aim of reducing the burden of COVID-19^5,6^.

Inactivated virus-based vaccine, BBIBP-CorV (Sinopharm), was among the first approved COVID-19 vaccines with successful safety profile. BBIP-CorV was the first administrated vaccine in Iran which the vast majority of the population were primed with it^7,8^. Although strong humoral immune response induction was seen in primary results, later studies showed that protective antibodies are not durable in some individuals after two doses of vaccination^9,10^.

Moreover, the emergence of the Omicron variant, has brought serious concerns regarding vaccine effectiveness and antibody persistency. Antibody fading have been reported after COVID-19 vaccination leading to reduced protection against both infection and hospitalization^11–14^.

In order to restore the immune responses, booster injections have been recommended though the optimal interval, dose and strategy are being explored. A booster dose could be administrated in a homologous or heterologous regimen^15–17^. In case of COVID-19, heterologous boosting has been recommended by some studies proposing that mix-and-match strategy would elicit and recover the immune responses better than the homologous agent. Owing to recent published data, a booster dose of a different type like mRNA or vector-based vaccines are more sufficient and could strongly induce specific antibodies against the virus^17–19^.

PastoCovac (Soberana 02) is manufactured in Pasteur Institute of Iran in collaboration with Finlay Vaccine Institute of Cuba. It is a recombinant protein vaccine composing a highly immunogenic region of SARS-CoV-2 Spike (RBD) conjugated to the tetanus toxin^20^. PastoCovac Plus (Soberana Plus) is also the booster dose of the candidate vaccine [dimer of RBD (50 µg)]^20–22^. The safety and immunogenicity of both vaccines as the priming and boosting doses were excellent^20,21^.

PastoCovac as the primary vaccine dose has the approval to be applied against SARS-CoV-2 with a high immunogenicity. The most commonly used vaccine against COVID-19 in Iran has been BBIBP-CorV. According to the need for administration of an optimum booster, identifying the booster vaccine which could provide safely stronger immunogenicity profile is important.

Hereby, we aimed at evaluation of immunogenicity and persistency of protein subunit vaccines (PastoCovac/Plus) and BBIBP-CorV as booster doses in Iranian population who were primarily vaccinated with two doses of BBIBP-CorV.

## Results

### Participants

Totally, 214 volunteers were evaluated including 108 males and 106 females. Twenty-five of the participants had at least one underlying disease and 36 individuals had a COVID-19 history (Table 1). There was no significant difference between BBIBP-CorV, PastoCovac and PastoCovac Plus booster groups in terms of mean age, sex and COVID-19 history (P > 0.05).

**Table 1:** Demographic and baseline characteristics of the participants.

Feature	BBIBP-CorV	PastoCovac Plus	PastoCovac	P-value
Population	(n = 74)	(n = 72)	(n = 68)	
Female	37 (50.0%)	35 (48.6%)	34 (50.0%)	0.982^b^
Male	37 (50.0%)	37 (51.4%)	34 (50.0%)	
Age				
Mean (SD)	43.0* (14.6)	41.9 (12.8)	41.3 (13.8)	0.775^a^
≤ 50	49 (66.2%)	53 (73.6%)	48 (70.6%)	0.618^b^
> 50	25 (33.8%)	19 (26.4%)	20 (29.4%)	
COVID history				
No	60 (81.1%)	63 (87.5%)	55 (80.9%)	0.484^b^
Yes	14 (18.9%)	9 (12.5%)	13 (19.1%)	
Underlying disease				
No	62 (83.8%)	65 (90.3%)	62 (91.2%)	0.320^b^
Yes	12 (16.2%)	7 (9.7%)	6 (8.8%)	

*SD* standard deviation.

^a^One-way ANOVA test, ^b^Chi-square test.

*A *P* value > 0.05 was considered significant.

The mean (SD) of the days since the last BBIBP-CorV (the 2nd dose) to get the booster shot (the 3rd dose) was 144.4 (28.1) for BBIBP-CorV/BBIBP-CorV group and 137.8 (25.4) for the both heterologous groups which did not show any significant difference between the three groups (*P: 0.39*).

### Immunogenicity evaluation

Anti-SARS-CoV-2 antibodies were evaluated 21 days after the booster shot. The specific antibodies were tracked and the geometric mean of antibodies’ titer, fold rise and also fourfold rise were compared with the obtained values before the injection.

**Figure 1:**
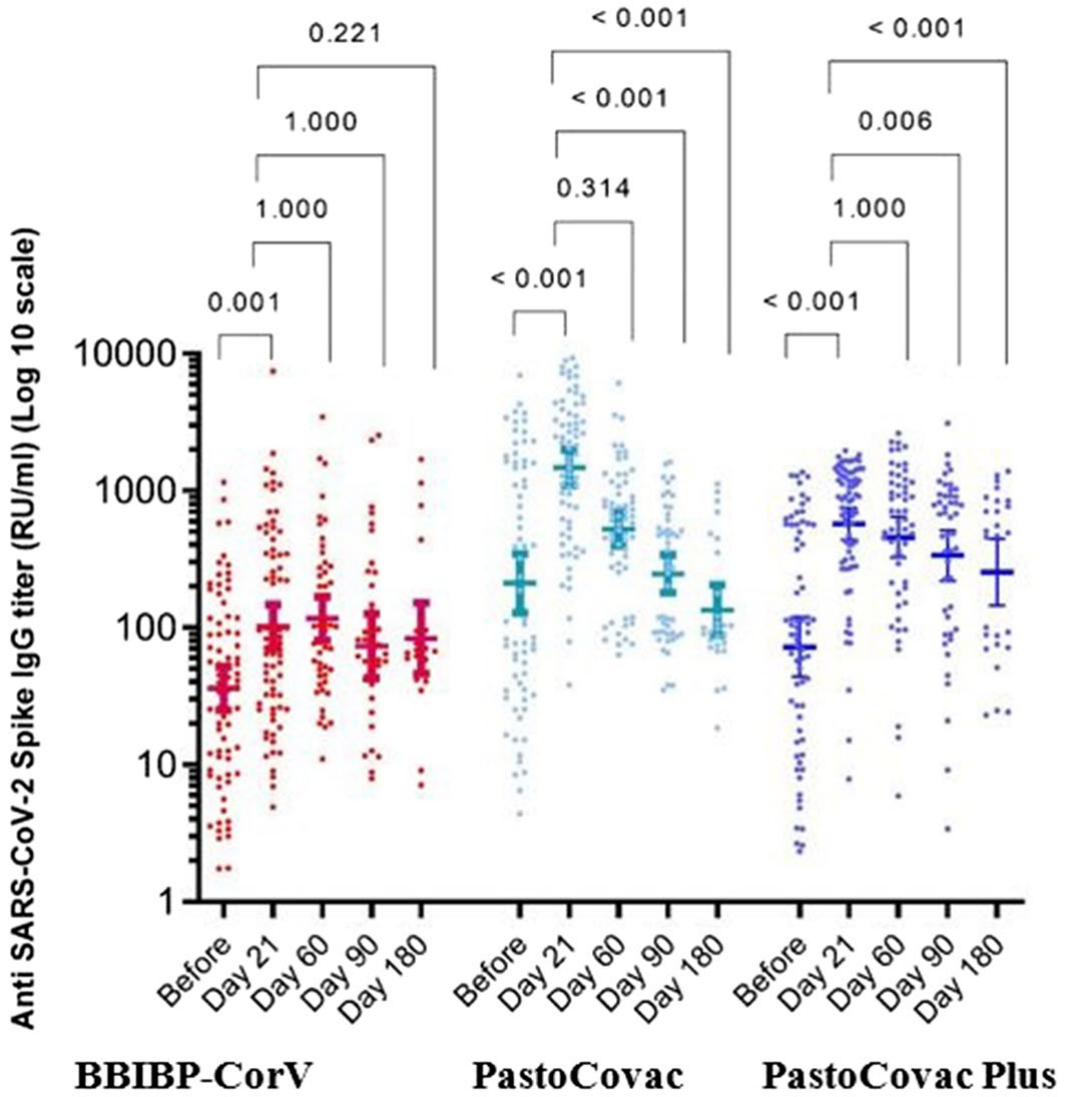
Anti-SARS-CoV-2 Spike antibody titers before and after injection of the three boosters during 6-month follow-up. Median and 95% confidence intervals are shown on the diagram. P-values were obtained using pairwise for Friedman test. Asymptotic significances (2-sided tests) are displayed, with a type I error of 0.05. Significance values have been adjusted for multiple comparisons using Bonferroni correction.

**Figure 2:**
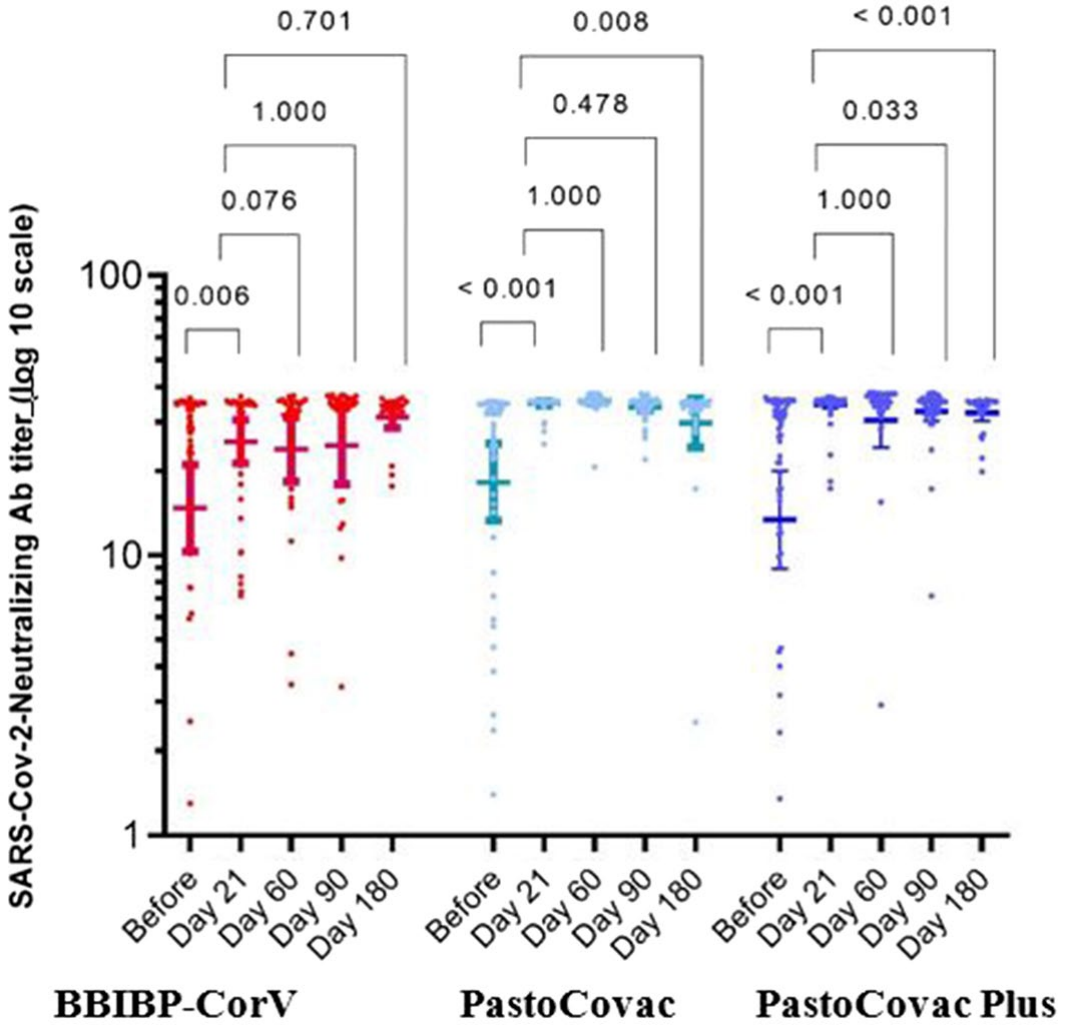
Anti-SARS-CoV-2 Neutralizing antibody titers before and after injection of the three boosters during 6-month follow-up. Median and 95% confidence intervals are shown on the diagram. P-values were obtained using pairwise for Friedman test. Asymptotic significances (2-sided tests) are displayed, with a type I error of 0.05.

**Figure 3:**
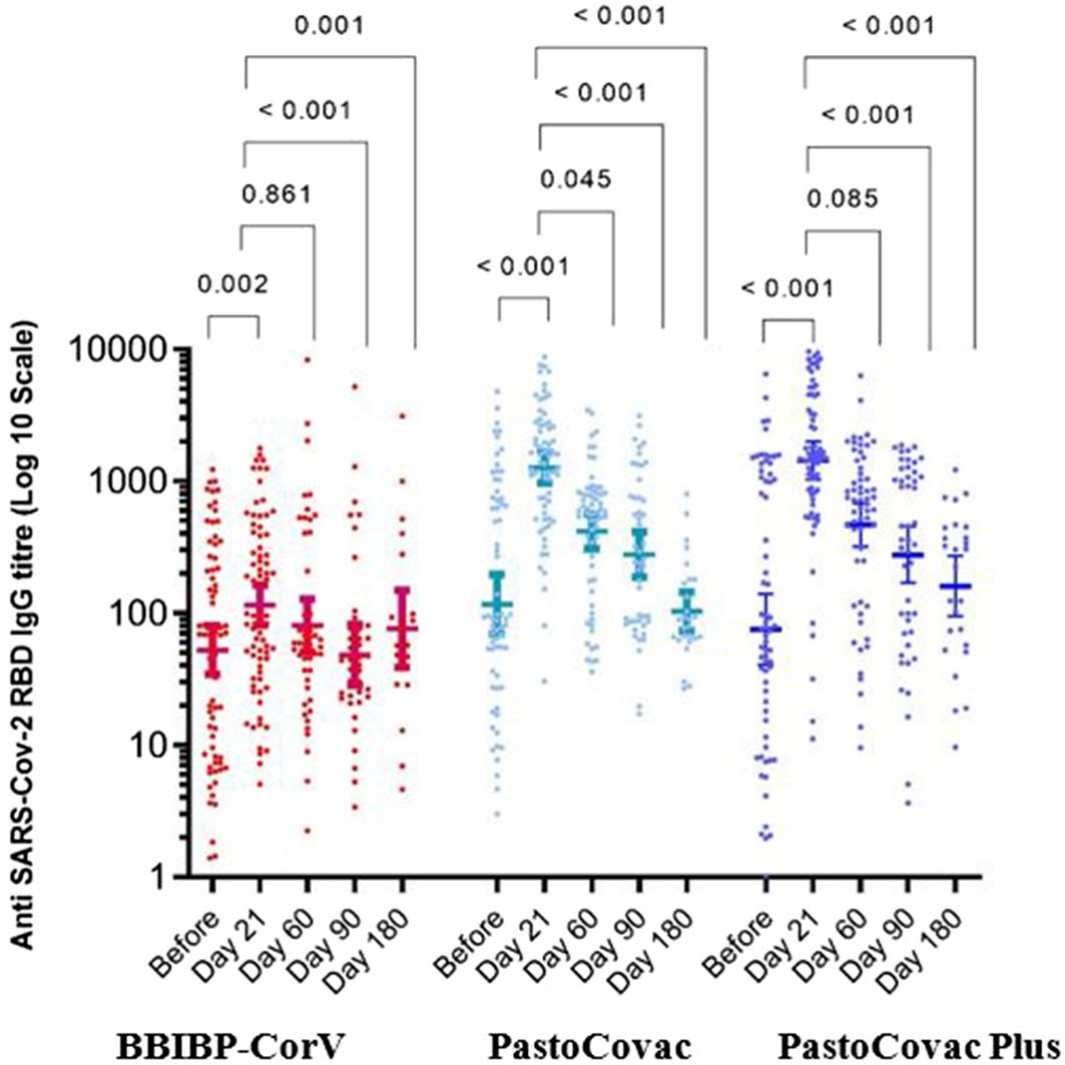
Anti-SARS-CoV-2 antibody titers before and after injection of the three boosters during 6-month follow-up. Median and 95% confidence intervals are shown on the diagram. P-values were obtained using pairwise for Friedman test. Asymptotic significances (2-sided tests) are displayed, with a type I error of 0.05. Significance values have been adjusted for multiple comparisons using Bonferroni correction.

All the seronegative individuals became seropositive after the booster shot of any type on day 21. PastoCovac booster recipients reached the highest anti-Spike IgG titer rise [717.0 (95% CI 485.4–1059.3)] among whom a fourfold rise of 50% was achieved (Table 2, Fig. 1.). Neutralizing and anti-RBD IgG antibody mean rise and fold rise were almost similar between the PastoCovac and PstoCovac Plus booster receivers (Table 2, Figs. 2 and 3). In contrast, BBIBP-CorV booster led to the lowest antibody mean rise, fold-rise and four-fold rise regarding anti-Spike, neutralizing and anti-RBD Abs (Figs. 1, 2 and 3).

**Table 2:** Evaluation of elicited antibodies against ARS-CoV-2 after the booster shots on day 21.

Antibody’s feature	BBIBP-CorV	PastoCovac Plus	PastoCovac	P-value
Anti-spike IgG				
Titer rise GMT (95% CI)	32.8 (20.2, 53.3)	352.6 (261.5, 475.5)	717.0 (485.4, 1059.3)	** < 0.001**^**a**^
Fourfold rise (n %)	13 (24.3%)	42 (63%)	32 (50%)	** < 0.001**^**b**^
Fold rise GMT (95% CI)	2.5 (1.9, 3.3)	8.7 (6.1, 12.5)	7.5 (4.8, 11.6)	** < 0.001**^**a**^
Neutralizing Ab				
Titer rise GMT (95% CI)	0.9 (0.5, 1.5)	3.7 (2.3, 6.0)	2.9 (1.8, 4.6)	**0.004**^**a**^
4-old rise (n %)	2 (3.2%)	13 (19.7%)	11 (17.5%)	0.261^b^
Fold rise GMT (95% CI)	1.3 (1.1, 1.5)	2.8 (1.8, 4.2)	2.0 (1.4, 2.7)	0.054^a^
Anti-RBD				
Titer rise GMT (95% CI)	31.0 (20.5, 46.7)	916.3 (614.0, 1367.5)	816.1 (599.3, 1111.4)	** < 0.001**^**a**^
Fourfold rise (n %)	10 (16.1%)	48 (72%)	39 (61.9%)	** < 0.001**^**b**^
Fold rise GMT (95% CI)	2.1 (1.6, 2.7)	21.5 (12.5, 37.3)	12.2 (7.5, 20.0)	** < 0.001**^**a**^

*GMT* geometric mean titer, *CI* confidence interval.

^a^Kruskal-Wallis test, ^b^Chi-square test.

Bold P values are indicated statistically significant.

### Long-term humoral immunity assessment

The persistency of generated specific antibodies against SARS-CoV-2 was tracked through three different window times which were compared to time before the booster injection and 21 days after it (Table 3 and Figs. 1, 2 and 3).

**Table 3:** SARS-CoV-2 specific antibodies titer trend during the 6-month follow-up.

Vaccine group (booster type)	Before	Day 21	Day 60	Day 90	Day 180	P-value^b^
Anti SARS-CoV-2 spike antibody (geometric mean, 95% CI)						
BBIBP-CorV	36.1 (25.1, 51.9)	101.9 (70.4, 147.3)	117.0 (81.2, 168.4)	73.5 (42.7, 126.6)	83.7 (46.2, 151.5)	** < 0.001**
PastoCovac-Plus	72.3 (43.8, 119.4)	574.2 (438.5 , 751.9)	457.0 (325.0, 642.7)	337.0 (219.9, 516.4)	253.3 (144.7, 443.5)	** < 0.001**
PastoCovac	210.7 (128.6, 345.3)	1,475.6 (1,092.4 , 1,993.3)	524.7 (397.9, 691.9)	247.3 (179.4, 340.9)	134.2 (88.1, 204.2)	** < 0.001**
P-value^a^	** < 0.001**	** < 0.001**	** < 0.001**	** < 0.001**	**0.012**	
Anti SARS-CoV-2 neutralizing antibody (geometric mean, 95% CI)						
BBIBP-CorV	14.8 (10.3, 21.1)	25.5 (21.3, 30.5)	23.9 (18.4, 31.1)	24.7 (18.0, 33.8)	31.3 (28.5, 34.2)	**0.001**
PastoCovac-Plus	13.4 (9.0, 20.1)	34.6 (33.5, 35.6)	30.4 (24.4, 37.9)	32.7 (30.3, 35.3)	32.3 (30.3, 34.4)	** < 0.001**
PastoCovac	18.3 (13.3, 25.0)	34.9 (34.4, 35.4)	35.6 (34.9, 36.4)	33.9 (32.9, 34.9)	29.7 (24.3, 36.4)	** < 0.001**
P-value^a^	0.648	** < 0.001**	** < 0.001**	0.344	0.703	
Anti SARS-CoV-2 RBD antibody (geometric mean, 95% CI)						
BBIBP-CorV	52.9 (34.7, 80.7)	115.6 (81.4, 164.0)	81.1 (51.3, 128.4)	48.3 (28.5, 81.9)	76.5 (38.9, 150.1)	** < 0.001**
PastoCovac-Plus	75.9 (41.0, 140.4)	1432.7 (1,029.6 , 1993.8)	465.7 (319.4 , 679.1)	277.2 (170.0, 452.0)	160.8 (94.9, 272.6)	** < 0.001**
PastoCovac	117.3 (69.9 ,196.6)	1259.6 (962.4, 1,648.6)	415.8 (308.3 , 560.9)	278.6 (188.8, 411.2)	103.5 (74.1, 144.6)	** < 0.001**
P-value^a^	0.055	** < 0.001**	** < 0.001**	** < 0.001**	0.081	

*CI* confidence interval.

^a^Kruskal-Wallis test, ^b^Friedman test.

Bold P values are indicated statistically significant.

Although the antibodies’ titers declined over the time, they were still in an acceptable level of persistency during the 6 months. According to BA.5 variant peak in Iran in this window time, a high rate of infection was recorded especially among BBIP-CorV vaccinated individuals. This led to an increase in antibody titers among BBIP-CorV group on day 180 which indicates the higher rate of COVID-19 incidence in comparison with PastoCovac/Plus groups though the confirmed COVID-19 infected cases with an episode of infection from day 90 to day 180 were excluded from the associated analysis. Neutralizing antibody showed better stability in comparison with anti-Spike and anti-RBD IgGs over the time.

Furthermore, upon adjustment of variables including sex, age, COVID-19 history and underlying disease, the median of anti-Spike IgG was higher in PastoCovac and PastoCovac Plus than BBIP-CorV on days 60, 90 and 180 (Supplementary data).

Comparison of neutralizing antibody titer was done on days 21, 60, 90 and 180 and it was found that the baseline level of neutralizing antibody on day 180 in individuals still has a higher level than day zero. (Table 3, Fig. 2). The mean titer of neutralizing antibody on day 180 is similar in all three groups. There was no significant difference in the reduction of neutralizing antibody between the three groups during 90–180 days after the booster shot.

### Neutralization assay

**Figure 4:**
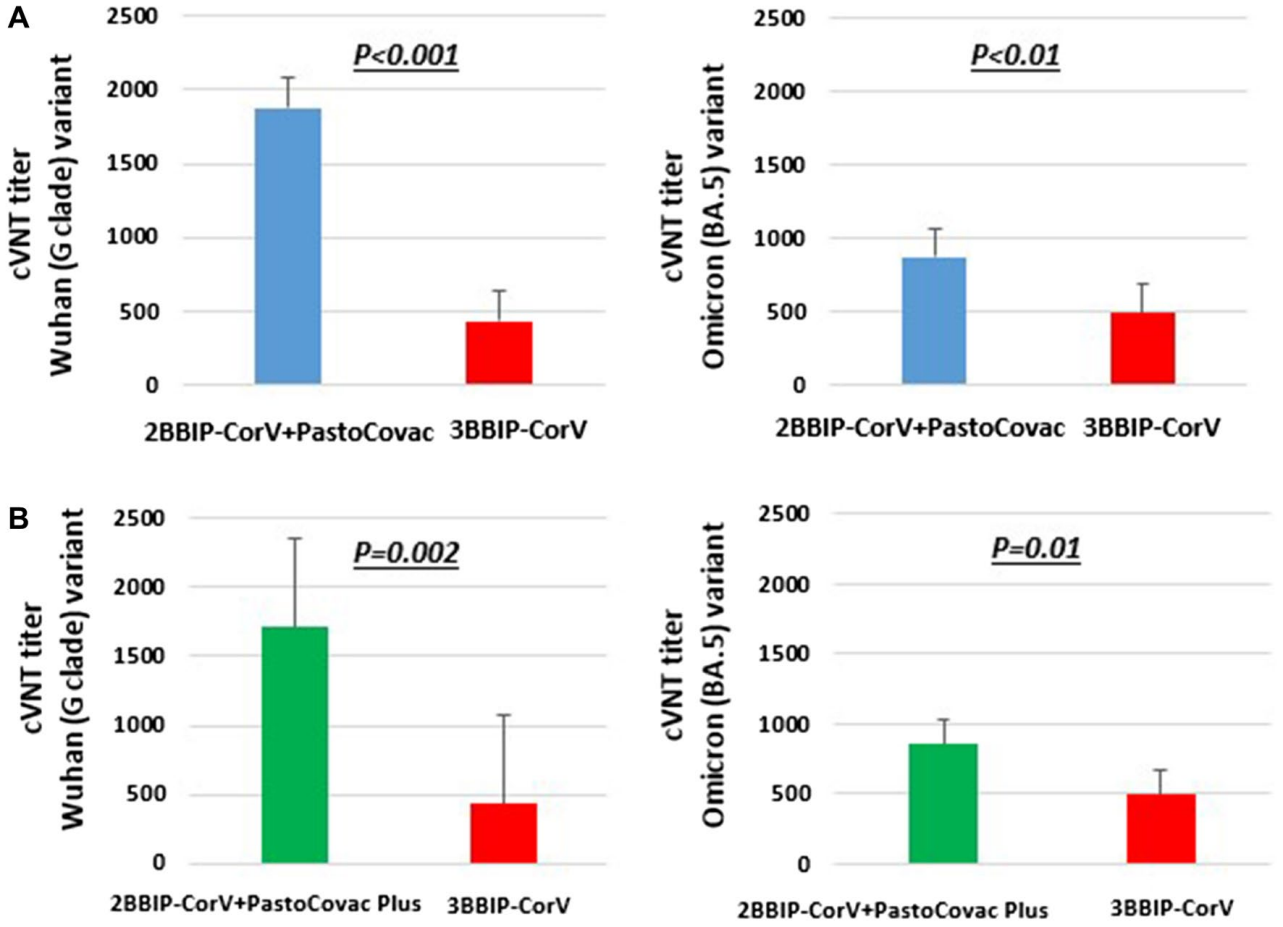
Comparison of virus neutralizing test between the applied boosters. Sera from 18 vaccinated individuals with complete schedule were evaluated (cVNT50: GMT, 95% CI) against variants Wuhan G clade and Omicron BA.5 variant. P values represent the statistic differences. (**A**) Two doses BBIBP-CorV + one dose PastoCovac; (**B**) Two doses BBIBP-CorV + one dose PastoCovac Plus in comparison with three doses of BBIBP-CorV.

The virus neutralization potency of the induced antibodies was assessed through cVNT50 test on 18 randomly selected sera samples against Wuhan and Omicron variants. The results showed that after PastoCovac booster dose, significant increases were observed against Wuhan (P < 0.001) and Omicron variant (P < 0.01) compared to homologous BBIBP-CorV regimen (Fig. 4A). In line with this, PastoCovac Plus also showed significantly higher neutralization potency than BBIBP-CorV shot against the both clades (Fig. 4B).

### Adverse events

No side effect was recorded within 30 min post the booster injections of any type. The safety assessment was done on day 7 through the phone call which showed that pain at the injection site was the only local event in 15.9%, 11.3% and 4.5% of the participants in PastoCovac Plus, BBIBP-CorV and PastoCovac groups, respectively. Furthermore, the systemic adverse events were observed in 19.4% of BBIBP-CorV, 14.3% of PastoCovac and 4.5% of PastoCovac Plus recipients among which headache and weakness were the most common systemic adverse events. There was no recorded adverse event on day 21 during the period of 3 weeks after the injection.

## Discussion

Traditional vaccine strategies were primarily developed applying the attenuated or inactivated whole virus particles against COVID-19. However, later studies demonstrated that this method does not provide the preferable protection^14,23^. Subunit vaccines based on the selected fragments have the potency to elicit antibody responses by only carrying the required antigenic components of the virus^24,25^.

PastoCovac as a protein subunit and primary series vaccine has shown great results in the clinical trials^26,27^. The present study shows excellent immunogenicity and the persistency of the induced antibodies of this vaccine as a booster shot in primarily vaccinated individuals with a 2-dose of BBIBP-CorV. The assessment of the antibody level on the first 21-day proved that PastoCovac is highly immunogenic through which fold rises of 7.5, 12.2 and 2.9 was achieved regarding SARS-CoV-2 anti-Spike, anti-RBD and neutralizing antibodies, respectively. Although the results of the seronegative individuals showed that the three applied boosters led to a 100% seropositivity, the four-fold rise regarding anti-Spike, neutralizing and anti-RBD antibodies were different between the homologous group and heterologous ones which highlights the great humoral immunity induction of PastoCovac and PastoCovac Plus as booster shots on an inactivated vaccine. Furthermore, the induced neutralizing antibodies examination proved that PasoCovac/Plus significantly neutralized SARS-CoV-2 variants.

In a study, NVSI-06-07 (recombinant protein-based vaccine) was evaluated as a booster dose in BBIBP-CorV vaccinated participants in parallel with BBIBP-CorV/BBIBP-CorV as the controls. The results showed that the induction of neutralizing antibodies against SARS-CoV-2 was higher in the protein-based booster receivers on day 28^28^. In our study, the neutralizing antibody titer rise in PastoCovac and PastoCovac Plus groups was 2.9 and 3.7 whereas in BBIBP-CorV with a rate of 0.9 on day 21. What is more, the highest rate of anti-Spike titer in PastoCovac receivers 717.0 followed by PastoCovac Plus 352.6 which is comparable with BBIBP-CorV recipients 32.8. In addition, anti-RBD titer rise was calculated as 916.3, 816.1 and 31.0 in PastoCovac Plus, PastoCovac and BBIBP-CorV groups.

The study from Cuba also showed that two doses of SOBERANA 02 (PastoCovac) combined with SOBERANA Plus (PastoCovac Plus) increased neutralizing antibodies which were detectable up to 8 months after the third dose. Moreover, sera samples of the vaccinated individuals neutralized the variants of concerns including Alpha, Beta, Delta, and Omicron^29^. The neutralization assay in our study also showed that both PastoCovac and PastoCovac Plus boosters neutralized Wuhan clade and also Omicron variant significantly more powerful than BBIBP-CorV.

Optimal boosting strategies are supposed to induce specific antibodies which are durable in order to the long-term pandemic control. Therefore, the persistency of humoral immune response has also come to attention according to the SARS-CoV-2 new waves of infection. The secondary outcomes of the present study proved that all the boosters led to a persistent antibody induction 180 days post the injection. The heterologous patterns resulted in a higher antibody titer mean compared to BBIP–CorV/ BBIP–CorV group during the 6-month follow-up. Interestingly, an increasing trend of antibodies is observed in only BBIP–CorV group on day 180. Nevertheless, the case-following indicated that the rate of infection was more prevalent in this group.

Our results indicate that booster shots of a different vaccine type against COVID-19 could lead to strong humoral immune responses and protein vaccines have the advantage of flexibility in cooperating with other vaccine platforms especially inactivated virus based types. However, in this study the protein subunit vaccine, PastroCovac/Plus, was only evaluated as a booster dose on individuals who were primed with inactivated virus vaccine which could be considered as a limitation though on the other study Adeno-based vaccine has been also considered (under review data). Moreover, PastoCovac vaccine was not applied as a priming dose in the present study which might have stronger humoral immune response than BBIP-CorV primed population due to the previously conducted study which assessed this issue (under review data).

## Conclusion

Administration of the same or different booster vaccines against COVID-19 in vaccinated individuals with BBIBP-CorV resulted in an acceptable level humoral immunity against COVID-19, however, protein subunit boosters showed a higher level of antibody induction with a great potency of SARS-Cov-2 neutralization ability in the present study beside an excellent safety profile. PastoCovac protein subunit vaccine, as a main vaccine dose against SARS-CoV-2, is hereby presented highly immunogenic with no safety concern as a booster shot. In other words, PastoCovac and PastoCovac Plus have similar immunogenicity, and safety profile as a booster dose and could be applied alternatively. Furthermore, the durability of the elicited antibodies proved the high rate of the elicited antibodies after six months.

## Material and methods

### Study design

This study is a non-randomized, open-labeled parallel clinical trial performed on individuals of Pasteur Institute of Iran with a 180-day follow-up schedule. Eligible individuals aged from18 to 80 years with controlled underlying disease were enrolled in the study who had received their first two-dose of BBIBP-CorV vaccine (28 ± 5 interval), 3–6 months prior to the admission.

The exclusion criteria included pregnant or breast-feeding women; individuals who got any vaccines in addition to two doses of BBIBP-CorV, presenting SARS-CoV-2 symptoms, having fever at the time or in a week prior to the enrollment and those under the immunosuppressive treatment in one month before the trial.

The eligible volunteers were free to choose one of the three boosters as PastoCovac (25 µg of Tetanus toxin conjugated to RBD), PastoCovac Plus (50 µg RBD-dimer) or BBIBP-CorV (6.5 U of inactivated SARS-CoV-2 antigen).

The study was approved by the Iran National Committee for Ethics in Biomedical Research (ethics code number: IR.NREC.1400.020) and was registered at the Iranian Registry of Clinical Trials (IRCT20131221015878N4, 26/02/2022).

All the participants were provided with the informed consent form before enrollment and all the protocol was performed according to the Declaration of Helsinki (Fortaleza, 13th Oct, 2013).

### Sampling and antibody assessment

Blood samples were collected before the booster shot and also on days 21, 60, 90 &180 post vaccination at site. Upon serum isolation, assessment of the generated antibodies was investigated with titers of anti-Spike IgG [Anti-SARS-CoV-2 Quantivac ELISA (IgG) (Euroimmun, Lübeck, Germany)], anti-RBD IgG [Anti-SARS-CoV-2 RBD (IgG), IDEALTASHKHIS, Iran] and neutralizing antibody (SARS-CoV-2 Neutralizing Ab IDEALTASHKHIS, Iran).

### Virus neutralizing test

Conventional neutralizing antibody titers (cVNT50) was evaluated on randomly selected samples^30^. Briefly, the samples were inactivated at 56 °C for 30 min. Vero cells were seeded in DMEM containing 10% FBS. Serial dilution of sera samples was prepared. Then 50 µl of each serum was mixed with 50 µl of 100 TCID_50_ of SARS-CoV-2 (D clade and BA.5 variant) and incubated at room temperature for an hour. Next, the prepared mixture was added into the wells containing monolayers of Vero cells for 60 min incubation at 37 °C. The control wells were as a well without the mixture of serum and virus, one well without any serums, and one well without any cells. After the incubation time and supernatant removal, the cells were washed with DMEM. After 72 h of maintaining in DMEM at 37 °C, the CPE was assessed applying an inverted microscope and the titer of neutralizing antibodies was evaluated in accordance to the highest serum dilution in which the virus was neutralized in 50% of the wells.

### Statistical analysis

The level of antibodies was assessed before and after the injection by geometric mean and standard deviation. Moreover, fourfold increase in antibody concentration over the baseline antibody was also investigated.

In order to calculate the antibodies fold-rise, the titer of antibodies after the booster shot was divided by its titer before the injection. The geometric mean of the calculated numbers was determined. In addition, antibody titer after the booster shot minus the antibody titer before the booster shot was calculated to achieve the pure titer of the antibody rise.

One way variance analysis and chi-squared test were applied to assess the variables including age, gender and COVID-19 history.

Chi-squared test was applied to compare the four-fold rise between the three groups. Friedman test was applied to assess the antibodies’ titer over the time. In addition, Logistic Regression models and Generalized Estimating Equations were used to assess the impact of the vaccines on the antibodies’ induction.

In each study arm, the frequency and frequency percentage of the solicited and unsolicited adverse events were calculated applying Chi-squared test. P values < 0.05 was considered statistically significant.

### Outcomes

The immunogenicity assessment was done regarding anti-SARS-CoV-2 RBD IgG, anti-SARS-CoV-2 Spike IgG and neutralizing antibodies between the three vaccine types on days 0, and 21. In addition, the safety assessment of the booster doses was also evaluated 30 min after the booster injection, on day 7 (via a phone call) and day 21 (at the site) regarding solicited systemic and local adverse events. The persistency of humoral immune responses were followed on days 60, 90 and 180.

## Supplementary Information

Supplementary Tables.

## Data Availability

Data that support the findings of this study are available upon reasonable request to the corresponding author.
