# Supplementary material for: PastoCovac and PastoCovac Plus as protein subunit COVID-19 vaccines led to great humoral immune responses in BBIP-CorV immunized individuals
Source: Sci Rep. 2023 May 18;13:8065. doi: 10.1038/s41598-023-35147-y (PMC10195798; doi:10.1038/s41598-023-35147-y)
Supplement: Supplementary file 1 — Supplementary Tables. [file 41598_2023_35147_MOESM1_ESM.docx]

Supplementary Table 1. Generalized estimating equations (GEE) of Anti-Spike Ab (QuantiVac) on day 180.

| QuantiVac (Spike) | Coef. | SE | P-Value | 95% CI | |
| --- | --- | --- | --- | --- | --- |
|  |  |  |  | Lower | Upper |
| Quantivac Before | 0.00 | 0.06 | 0.989 | -0.11 | 0.11 |
| Vaccine type |  |  |  |  |  |
| Pastocovac-Plus | 429.15 | 145.33 | 0.004 | 139.07 | 719.23 |
| Pastocovac | 37.64 | 148.21 | 0.800 | -258.19 | 333.48 |
| Age | 0.09 | 5.08 | 0.985 | -10.04 | 10.23 |
| Sex (Male) | 4.52 | 108.59 | 0.967 | -212.23 | 221.26 |
| Covid history (Yes) | 49.45 | 144.36 | 0.733 | -238.69 | 337.60 |
| Underlying disease (Yes) | 7.24 | 171.29 | 0.966 | -334.65 | 349.14 |
| Constant | 55.41 | 208.25 | 0.791 | -360.27 | 471.08 |

By adjusting the effect of other variables and the value of day zero, it was found that the mean of Anti-Spike IgG on day 180 in the PastoCovac-Plus group is higher than the Sinopharm group, and this difference is significant. The mean Anti-Spike IgG on day 180 in the PastoCovac group is higher than the Sinopharm group, but it is not statistically significant.

| Supplementary Table 2. Generalized estimating equations (GEE) of Anti-Spike Ab (QuantiVac) on day 90. | | | | | |
| --- | --- | --- | --- | --- | --- |
| QuantiVac (Spike) | Coef. | SE | P-Value | 95% CI | |
|  |  |  |  | Lower | Upper |
| Quantivac Before | 0.02 | 0.06 | 0.743 | -0.10 | 0.14 |
| Vaccine type |  |  |  |  |  |
| Pastocovac-Plus | 497.66 | 131.28 | 0.000 | 237.89 | 757.42 |
| Pastocovac | 143.77 | 136.07 | 0.293 | -125.46 | 413.01 |
| Age | 6.83 | 4.31 | 0.116 | -1.70 | 15.36 |
| Sex (Male) | -22.48 | 103.76 | 0.829 | -227.79 | 182.84 |
| Covid history (Yes) | 189.19 | 141.43 | 0.183 | -90.65 | 469.03 |
| Underlying disease (Yes) | -0.52 | 154.79 | 0.997 | -306.80 | 305.76 |
| Constant | -169.95 | 193.18 | 0.381 | -552.19 | 212.29 |

By adjusting the effect of other variables and the value of day zero, it was found that the mean of Anti-Spike IgG on day 90 in the PastoCovac-Plus group is higher than the Sinopharm group, and this difference is significant. The mean Anti-Spike IgG on day 180 in the PastoCovac group is higher than the Sinopharm group, but it is not statistically significant.

| Supplementary Table 3. Generalized estimating equations (GEE) of Anti-Spike Ab (QuantiVac) on day 60. | | | | | |
| --- | --- | --- | --- | --- | --- |
| QuantiVac (Spike)60 | Coef. | SE | P-Value | 95% CI | |
|  |  |  |  | Lower | Upper |
| Quantivac Before | -0.02 | 0.05 | 0.658 | -0.12 | 0.08 |
| Vaccine type |  |  |  |  |  |
| Pastocovac-Plus | 534.18 | 110.60 | 0.000 | 315.75 | 752.60 |
| Pastocovac | 504.37 | 115.80 | 0.000 | 275.68 | 733.07 |
| Age | 4.66 | 3.32 | 0.163 | -1.90 | 11.23 |
| Sex (Male) | 20.99 | 87.25 | 0.810 | -151.33 | 193.31 |
| Covid history (Yes) | 170.44 | 123.06 | 0.168 | -72.60 | 413.48 |
| Underlying disease (Yes) | -4.57 | 134.86 | 0.973 | -270.90 | 261.77 |
| Constant | -91.75 | 160.48 | 0.568 | -408.68 | 225.19 |

By adjusting the effect of other variables and the value of day zero, it was found that the mean of Anti-Spike IgG on day 60 in the PastoCovac-Plus group is higher and significant than the Sinopharm group. Also, the mean of Anti-Spike IgG on day 60 in the PastoCovac group is higher and significant than the Sinopharm group.

| Supplementary Table 4. Generalized estimating equations (GEE) of Anti-Spike Ab (QuantiVac) on day 21. | | | | | |
| --- | --- | --- | --- | --- | --- |
| QuantiVac (Spike) | Coef. | SE | P-Value | 95% CI | |
|  |  |  |  | Lower | Upper |
| Quantivac Before | 1.08 | 0.07 | 0.000 | 0.95 | 1.21 |
| Vaccine type |  |  |  |  |  |
| Pastocovac-Plus | 396.86 | 127.16 | 0.002 | 146.16 | 647.55 |
| Pastocovac | 763.20 | 139.05 | 0.000 | 489.05 | 1037.35 |
| Age | 0.34 | 3.97 | 0.931 | -7.48 | 8.17 |
| Sex (Male) | -7.06 | 104.56 | 0.946 | -213.20 | 199.08 |
| Covid history (Yes) | 161.64 | 141.59 | 0.255 | -117.52 | 440.79 |
| Underlying disease (Yes) | 6.13 | 168.21 | 0.971 | -325.52 | 337.77 |
| Constant | 3.81 | 195.35 | 0.984 | -381.34 | 388.96 |

By adjusting the effect of other variables and the value of day zero, it was found that the mean of Anti-Spike IgG on day 21 in the PastoCovac-Plus group was higher and significant than the Sinopharm group. Also, the mean of Anti-Spike IgG on day 21 in the PastoCovac group is higher and significant than the Sinopharm group.

| Supplementary Table 5. Generalized estimating equations (GEE) of Neutralizing Ab on day 180. | | | | | |
| --- | --- | --- | --- | --- | --- |
| Neutralizing Ab | Coef. | SE | P-Value | 95% CI | |
|  |  |  |  | Lower | Upper |
| Neut Before | 0.10 | 0.07 | 0.167 | -0.04 | 0.23 |
| Vaccine type |  |  |  |  |  |
| Pastocovac-Plus | 1.11 | 1.83 | 0.545 | -2.54 | 4.77 |
| Pastocovac | 1.03 | 1.80 | 0.569 | -2.56 | 4.62 |
| Age | -0.03 | 0.06 | 0.669 | -0.16 | 0.10 |
| Sex (Male) | 0.20 | 1.50 | 0.895 | -2.79 | 3.18 |
| Covid history (Yes) | 1.04 | 1.86 | 0.578 | -2.67 | 4.76 |
| Underlying disease (Yes) | -0.23 | 2.15 | 0.916 | -4.51 | 4.06 |
| Constant | 31.26 | 3.61 | 0.000 | 24.05 | 38.47 |

By adjusting the effect of other variables and the value of day zero, it was found that the average neutralizing Ab on day 180 in none of the PastoCovac-Plus and PastoCovac groups is higher than the Sinopharm group.

| Supplementary Table 6. Generalized estimating equations (GEE) of Neutralizing Ab on day 90. | | | | | |
| --- | --- | --- | --- | --- | --- |
| Neutralizing Ab | Coef. | SE | P-Value | 95% CI | |
|  |  |  |  | Lower | Upper |
| Neut Before | 0.02 | 0.04 | 0.664 | -0.06 | 0.09 |
| Vaccine type |  |  |  |  |  |
| Pastocovac-Plus | 0.96 | 1.03 | 0.353 | -1.08 | 2.99 |
| Pastocovac | 0.47 | 1.00 | 0.640 | -1.52 | 2.46 |
| Age | 0.02 | 0.03 | 0.467 | -0.04 | 0.09 |
| Sex (Male) | -0.30 | 0.84 | 0.723 | -1.97 | 1.37 |
| Covid history (Yes) | 0.14 | 1.13 | 0.902 | -2.09 | 2.37 |
| Underlying disease (Yes) | 0.20 | 1.21 | 0.866 | -2.18 | 2.59 |
| Constant | 32.93 | 1.87 | 0.000 | 29.22 | 36.63 |

By adjusting the effect of other variables and the value of day zero, it was found that the median neutralizing Ab on day 90 in none of the Pastocovac-Plus and Pastocovac groups is higher than the sinopharm group.

| Supplementary Table 7. Generalized estimating equations (GEE) of Neutralizing Ab on day 60. | | | | | |
| --- | --- | --- | --- | --- | --- |
| Neutralizing Ab | Coef. | SE | P-Value | 95% CI | |
|  |  |  |  | Lower | Upper |
| Neut Before | 0.04 | 0.03 | 0.153 | -0.02 | 0.10 |
| Vaccine type |  |  |  |  |  |
| Pastocovac-Plus | 4.15 | 0.87 | 0.000 | 2.44 | 5.87 |
| Pastocovac | 4.09 | 0.85 | 0.000 | 2.42 | 5.77 |
| Age | 0.01 | 0.03 | 0.707 | -0.04 | 0.06 |
| Sex (Male) | -0.29 | 0.70 | 0.679 | -1.68 | 1.09 |
| Covid history (Yes) | 1.72 | 1.02 | 0.093 | -0.29 | 3.72 |
| Underlying disease (Yes) | 0.30 | 1.06 | 0.778 | -1.79 | 2.39 |
| Constant | 29.89 | 1.53 | 0.000 | 26.88 | 32.91 |

By adjusting the effect of other variables and the value of day zero, it was found that the mean of neutralizing Ab on day 60 in the PastoCovac-Plus group is higher and significant than the Sinopharm group. Also, the mean of neutralizing Ab on day 60 in the PastoCovac group is higher and significant than the Sinopharm group.

| Supplementary Table 8. Generalized estimating equations (GEE) of Neutralizing Ab on day 21. | | | | | |
| --- | --- | --- | --- | --- | --- |
| Neutralizing Ab | Coef. | SE | P-Value | 95% CI | |
|  |  |  |  | Lower | Upper |
| Neut Before | 0.05 | 0.03 | 0.067 | 0.00 | 0.10 |
| Vaccine type |  |  |  |  |  |
| Pastocovac-Plus | 2.03 | 0.72 | 0.005 | 0.61 | 3.44 |
| Pastocovac | 1.48 | 0.73 | 0.043 | 0.04 | 2.91 |
| Age | 0.00 | 0.02 | 0.981 | -0.04 | 0.04 |
| Sex (Male) | -0.01 | 0.60 | 0.987 | -1.20 | 1.18 |
| Covid history (Yes) | 0.27 | 0.83 | 0.747 | -1.36 | 1.89 |
| Underlying disease (Yes) | 0.07 | 0.95 | 0.941 | -1.80 | 1.94 |
| Constant | 32.42 | 1.32 | 0.000 | 29.82 | 35.02 |

By adjusting the effect of the rest of the variables and the day 0 value, it was found that the mean of neutralizing Ab on day 21 in the PastoCovac-Plus group was higher and significant than the Sinopharm group. Also, the mean of neutralizing Ab on day 21 in the PastoCovac group is higher and significant than the Sinopharm group.

| Supplementary Table 9. Generalized estimating equations (GEE) of Anti-RBD Ab on day 180. | | | | | |
| --- | --- | --- | --- | --- | --- |
| Anti-RBD Ab | Coef. | SE | P-Value | 95% CI | |
|  |  |  |  | Lower | Upper |
| RBD Before | 0.01 | 0.03 | 0.658 | -0.04 | 0.07 |
| Vaccine type |  |  |  |  |  |
| Pastocovac-Plus | 243.96 | 81.43 | 0.004 | 81.43 | 406.50 |
| Pastocovac | 38.76 | 78.90 | 0.625 | -118.74 | 196.25 |
| Age | 0.58 | 2.80 | 0.837 | -5.01 | 6.17 |
| Sex (Male) | -10.63 | 59.49 | 0.859 | -129.38 | 108.12 |
| Covid history (Yes) | 42.32 | 79.13 | 0.595 | -115.62 | 200.25 |
| Underlying disease (Yes) | 55.21 | 93.34 | 0.556 | -131.09 | 241.52 |
| Constant | 24.46 | 115.32 | 0.833 | -205.73 | 254.64 |

By adjusting the effect of other variables and the day 0 value, it was found that the mean of anti-RBD IgG on day 180 in the PastoCovac-Plus group was higher and significant than the Sinopharm group. But the mean of anti-RBD IgG on day 180 in the PastoCovac group is not significantly different from the Sinopharm group.

| Supplementary Table 10. Generalized estimating equations (GEE) of Anti-RBD Ab on day 90. | | | | | |
| --- | --- | --- | --- | --- | --- |
| Anti-RBD Ab | Coef. | SE | P-Value | 95% CI | |
|  |  |  |  | Lower | Upper |
| RBD Before | 0.04 | 0.06 | 0.494 | -0.08 | 0.17 |
| Vaccine type |  |  |  |  |  |
| Pastocovac-Plus | 296.65 | 153.26 | 0.055 | -6.60 | 599.90 |
| Pastocovac | 180.50 | 149.53 | 0.230 | -115.37 | 476.37 |
| Age | 7.33 | 4.97 | 0.142 | -2.50 | 17.17 |
| Sex (Male) | -97.59 | 119.31 | 0.415 | -333.67 | 138.49 |
| Covid history (Yes) | 175.22 | 161.77 | 0.281 | -144.88 | 495.32 |
| Underlying disease (Yes) | 5.07 | 177.51 | 0.977 | -346.16 | 356.30 |
| Constant | -204.73 | 223.11 | 0.361 | -646.19 | 236.73 |

By adjusting the effect of other variables and the day 0 value, it was found that the difference in the mean RBD on day 90 is not significant in any of the PastoCovac-Plus and PastoCovac groups compared to the Sinopharm group.

| Supplementary Table 11. Generalized estimating equations (GEE) of Anti-RBD Ab on day 60. | | | | | |
| --- | --- | --- | --- | --- | --- |
| Anti-RBD Ab | Coef. | SE | P-Value | 95% CI | |
|  |  |  |  | Lower | Upper |
| RBD Before | 0.05 | 0.05 | 0.255 | -0.04 | 0.15 |
| Vaccine type |  |  |  |  |  |
| Pastocovac-Plus | 568.01 | 109.12 | 0.000 | 352.52 | 783.49 |
| Pastocovac | 460.77 | 107.03 | 0.000 | 249.41 | 672.13 |
| Age | 2.04 | 3.24 | 0.529 | -4.35 | 8.44 |
| Sex (Male) | -4.62 | 84.91 | 0.957 | -172.29 | 163.05 |
| Covid history (Yes) | 68.89 | 120.20 | 0.567 | -168.47 | 306.25 |
| Underlying disease (Yes) | 76.91 | 131.46 | 0.559 | -182.68 | 336.49 |
| Constant | -24.15 | 157.27 | 0.878 | -334.71 | 286.41 |

By adjusting the effect of other variables and the value of day zero, it was found that the mean of anti-RBD IgG on day 60 in the PastoCovac-Plus group was higher and significant than the Sinopharm group. Also, the median anti-RBD IgG on day 60 in the PastoCovac group is higher and significant than the Sinopharm group.

| Supplementary Table 12. Generalized estimating equations (GEE) of Anti-RBD Ab on day 21. | | | | | |
| --- | --- | --- | --- | --- | --- |
| Anti-RBD Ab | Coef. | SE | P-Value | 95% CI | |
|  |  |  |  | Lower | Upper |
| RBD Before | 1.10 | 0.08 | 0.000 | 0.94 | 1.25 |
| Vaccine type | 1066.35 | 157.95 | 0.000 | 754.95 | 1377.76 |
| Pastocovac-Plus | 971.34 | 158.98 | 0.000 | 657.90 | 1284.79 |
| Pastocovac | 0.13 | 4.83 | 0.978 | -9.40 | 9.66 |
| Age | 29.05 | 127.78 | 0.820 | -222.88 | 280.98 |
| Sex (Male) | 26.09 | 172.58 | 0.880 | -314.16 | 366.34 |
| Covid history (Yes) | -9.66 | 204.87 | 0.962 | -413.57 | 394.25 |
| Underlying disease (Yes) | 5.22 | 238.40 | 0.983 | -464.80 | 475.24 |
| Constant | 1.10 | 0.08 | 0.000 | 0.94 | 1.25 |

By adjusting the effect of other variables and the value of day zero, it was found that the mean of anti-RBD IgG on day 21 in the PastoCovac-Plus group was higher and significant than the Sinopharm group. Also, the mean of anti-RBD IgG on day 21 in the PastoCovac group is higher and significant than the Sinopharm group.
